# Tackling the Stability Issues of Silver Nanowire Transparent Conductive Films through FeCl_3_ Dilute Solution Treatment

**DOI:** 10.3390/nano9040533

**Published:** 2019-04-03

**Authors:** Xikun Chu, Ke Wang, Jingqi Tao, Shuxin Li, Shulin Ji, Changhui Ye

**Affiliations:** 1 Key Laboratory of Materials Physics, Anhui Key Laboratory of Nanomaterials and Nanotechnology, Institute of Solid State Physics, Chinese Academy of Sciences, Hefei 230031, China; xikunchu@163.com (X.C.); jqtao127@163.com (J.T.); lishuxin@issp.ac.cn (S.L.); 2 Science Island Branch of Graduate School, University of Science and Technology of China, Hefei 230026, China; 3 Key Laboratory of Silicon Device Technology, Institute of Microelectronics, Chinese Academy of Sciences, Beijing 100029, China; wangke@ime.ac.cn; 4 College of Materials Science and Engineering, Zhejiang University of Technology, Hangzhou 310014, China

**Keywords:** silver nanowires, chemisorption-related Fermi level shift, transparent conductive films, size-dependent instability, surface atomic diffusion blocking

## Abstract

Silver nanowires (AgNWs) have been investigated as alternatives to indium tin oxide in transparent conductive films (TCFs) for electronics. However, AgNW TCFs still pose stability issues when exposed to thermal, chemical, and mechanical stimuli. Herein, we demonstrate a facile and effective route to improve stability by treating the films with dilute ferric chloride solution. Our results indicate that after treatment the films exhibit a dramatically enhanced stability against aging, high temperature oxidation, chemical etching, sulfurization, and mechanical straining. Size-dependent instability is fully explored and explained regarding surface atomic diffusion, which could be blocked by enhancing the activation energy of surface diffusion through forming a AgCl cap under ferric chloride solution treatment. Chemisorption-related Fermi level shift of silver nanowires is applied to tune their chemical reactivity to ferric chloride solution for balancing between size-dependent stability improvement and maintaining optoelectrical properties. Owing to the dilute treatment solution, the treated films exhibit a negligible change in light transmittance, whereas sheet resistance decreases by 30% and flexibility increases because of capillary-force-induced welding of contacting AgNWs and AgCl layer mediated tightening. These findings are significant for real-world applications of AgNW TCFs.

## 1. Introduction

Currently, the most commonly used transparent conductive films (TCFs) in the electronic industry are based on indium tin oxide (ITO). Its excellent optical transparency and the low sheet resistance have extended the use of ITO as electrodes in highly demanding applications [1]. However, ITO has some inherent drawbacks, such as an inconvenient deposition process, relatively high cost, and brittle nature [2], limiting its widespread applications. Several alternative TCFs have been proposed, such as carbon nanotubes (CNTs) [3,4,5], graphene [6], conducting polymers [7], and metallic nanostructures [8,9,10]. Among them, silver nanowire (AgNW) TCFs exhibit the most promising performance with a low sheet resistance of ~10 Ω/sq and a high optical transmittance of ~90% [11]. In addition, AgNWs can be dispersed as an ink [12,13,14,15] and readily deposited onto arbitrary substrates using simple solution-coating techniques, rendering AgNW TCFs particularly attractive for use in various optoelectrical devices. Solar cells [16], light emitting devices [17,18], transparent thin-film heaters [19,20], smart windows [21,22], and touch screens [23] have already been demonstrated by employing AgNW TCFs.

Despite the above-mentioned advantages of AgNWs, they have not been well received by the market due to their stability issues. AgNW TCFs are easily oxidized in moist air due to their interactions with moisture, oxygen, and sulfur-containing compounds [24]. In addition, AgNW TCFs have limited thermal stability due to the Rayleigh instability phenomenon when they are thermally annealed at approximately 200 °C for tens of minutes [25,26]. Besides, AgNWs tend to delaminate from the substrate under a bending strain. To address the stability issues, some effective approaches have been proposed. For example, AgNWs were embedded in polymer to improve the chemical stability [27,28]; however, sulfur-containing gases could still diffuse though small voids or cracks in the polymer layer and oxidize AgNWs [28]. It was demonstrated that AgNWs protected by graphene oxide (GO) sheets [29,30], Ni, and/or Au metals [31,32] exhibited better chemical stability in air. Unfortunately, GO or metal layers on AgNW TCFs reduced the transmittance to some extent. Introducing SiO_2_ into AgNW TCFs can enhance the thermal stability of AgNWs without sacrifice of their optoelectrical performance; however, SiO_2_ is a typical acidic oxide, which is not suitable for use in an alkaline environment [33]. Atomic layer deposition of ZnO [18], Al_2_O_3_ [34], or TiO_2_ [35] on AgNW TCFs has been shown to increase the thermal stability as well as the mechanical reliability; nevertheless, it requires a specific apparatus, and the cost is high for industrial production. These methods are relatively complicated and somewhat negatively affect the overall optoelectrical performance of AgNW TCFs. It is still a challenge to develop an efficient method which can be applied under mild conditions to improve the stability of the AgNW TCFs without sacrificing the optoelectrical performance. Another key issue which needs attention is the size-dependent stability of AgNWs, as thin or thick nanowires are required for different purposes. For example, ultrathin AgNWs are optimal for TCFs with a low haze in high definition touch panel displays, while thick ones are better for TCFs with a large haze and low sheet resistance in solar cells [15,36]. Up until now, little systematical work [37,38,39] has been done on the mechanism and inhibition of size-dependent instability of AgNW TCFs.

In this paper, we report a method of improving the stability of AgNW TCFs through FeCl_3_ dilute solution (FeCl_3_-DS) treatment. Compared with other methods, this solution process does not require any external energy or complex equipment and brings no negative effect to the optoelectrical performance of AgNW TCFs. Instead, the FeCl_3_-DS treated AgNW TCFs exhibit 30% lower sheet resistance caused by the capillary force [37,40], and a negligible change in light transmittance. It is noteworthy that aging, thermal, and chemical stabilities as well as mechanical flexibility are meanwhile much improved in the FeCl_3_-DS treated AgNW TCFs. Using thermal stability as an example, the relationship between the safe working temperature of TCFs and the AgNW diameter is systematically studied and different FeCl_3_-DS treatments are employed to simultaneously enhance the safe working temperature and balance the optoelectrical performance for AgNW TCFs of different nanowire diameters. Our studies demonstrate that this simple and low-power-consumption method provides an innovative approach for preparing AgNW TCFs for real-world applications

## 2. Materials and Methods

### 2.1. Materials

Silver nitrate (AgNO_3_, ≥99.8%) was purchased from Shanghai Qiangshun Chemical Reagent Co., Ltd., Zhabei, SH, China). Ferric chloride (FeCl_3_, Sinopharm Chemical Reagent Co., Ltd., Jingan, SH, China), ethylene glycol (EG, Sinopharm Chemical Reagent Co., Ltd., Jingan, SH, China), polyvinylpyrrolidone (PVP-55000, average molecular weight of 55,000, and PVP-360000, Sinopharm Chemical Reagent Co., Ltd., Jingan, SH, China) were used as-received without further purification. Cleaned polyimide (PI) and polyethylene terephthalate (PET) were used as flexible transparent substrates.

### 2.2. Synthesis of AgNW

#### 2.2.1. AgNWs with an Average Diameter of 20 nm

The synthetic method was based on previous reports by our group with minor modifications [41,42]. NaBr solution (0.0114 g in 0.5 mL of EG), NaCl solution (0.0123 g in 1 mL of EG), PVP-360000 solution (0.2800 g in 5 mL of EG), and fresh AgNO_3_ solution (0.2255 g in 5 mL of EG) were individually prepared for use. All solutions were added into a 100 mL flask with 38.5 mL EG placed in an oil bath at room temperature. The mixed solution was moderately stirred for 30 min, and then slowly heated to 180 °C in 10–20 min, during which time the nitrogen gas with a flux of 150 mL/min was bubbled into the solution. Once the temperature reached 180 °C, the solution was naturally cooled to 160 °C without stirring. After a 2 h reaction at 160 °C without disturbances, the solution was cooled to room temperature. The synthetic solution was washed and AgNWs purified to remove large AgCl and AgBr particles and small Ag particles. Then, the purified AgNWs with high aspect ratios (>1000) were obtained with an average diameter of 20 nm and length of 34 μm, respectively. Finally, ethanol solution of dispersed AgNWs was prepared for film coating.

#### 2.2.2. AgNWs with an Average Diameter of 50 nm

Thicker AgNWs of 50 nm were synthesized by the mixed PVP technique of different molecular weights, which was modified from our earlier report [43]. A mixture of PVP (0.421 g PVP-55000 and 0.406 g PVP-360000) was dissolved in 115 mL of EG, and heated to 140 °C. Then, 12.5 mL of FeCl_3_ solution (1.1 mM in EG) and 20 mL of AgNO_3_ solution (0.9 g dissolved in EG by sonication in an ice bath for 5 min) were added in sequence into the PVP solution within 4 min. The reaction was performed at 140 °C for 55 min, after which the solution was cooled and filtrated. The remaining AgNW precipitate was dispersed into ethanol and centrifuged at the speed of 2000 rpm three times to remove EG, PVP, and other impurities. Finally, uniform AgNWs were dispersed in ethanol.

#### 2.2.3. AgNWs with an Average Diameter of 100 nm

The synthetic method of producing 100 nm AgNWs was similar to that of the 50 nm AgNWs, except for the slowing down of the injection speed of FeCl_3_ and AgNO_3_ solution from an injection time of 4 min to 11 min.

### 2.3. Formation of AgNW TCFs and Treatment with FeCl_3_ Dilute Solutions

AgNW TCFs were prepared by an automatic coating machine (BEVS 1811/2) equipped with a Meyer rod (OSP-25). AgNWs were coated on PI or PET substrates with a coating rate of 180 mm/s. TCFs were obtained after a brief drying process at 80 °C for 5 min. FeCl_3_-DS with a concentration of 0.007–0.14 mM (1.12–22.4 × 10^−4^ wt %) was prepared by dissolving FeCl_3_ in deionized water. AgNW TCFs were immersed in FeCl_3_-DS for different duration time, followed by washing with deionized water and drying under ambient conditions.

### 2.4. Characterization

Scanning electron microscopy (SEM) (Sirion 200 FEG, FEI Co., Ltd., Hillsborough, OR, USA.) and transmission electron microscopy (TEM) (JEM-2010, JEOL Co., Ltd., Akishima, TKY, Japan) coupled with energy dispersive X-ray spectroscopy (EDS) were used to characterize the morphologies of AgNWs and TCFs. Optical transmittance spectra were obtained on an UV-vis-NIR spectrometer (SolidSpec-3600, Shimadzu Co., Ltd., Kyoto, KF, Japan). Sheet resistances were measured by a four-point probe technique. A four-point probe composed of four points in an equidistant linear array equipped on the apparatus (RTS-9, Four-Probe Tech, Guangzhou Four Probe Technology Co., Ltd., Tianhe, GZ, China) was in close contact with the AgNW TCFs. When a constant low-value current flowed through the outer two points, the voltage drop between the inner two points was recorded and calculated sheet resistance values were shown on the apparatus display. X-ray photoelectron spectra (XPS) were recorded using an electron spectrometer fit with an Al Kα source (Thermo ESCALAB 250, soft X-ray source at 1486.6 eV, ThermoFisher Scientific Co., Ltd., Waltham, MA, USA). The aging test was conducted by exposing AgNW TCFs to the lab atmosphere. We recorded the results every 3 days for 60 days. The thermal stability was checked by annealing the PI in a tube furnace in air, whose sheet resistance was measured when the temperature was raised up every 25 °C. The chemical stability was tested by immersing the TCFs into various chemical solutions. A H_2_S etching test was carried out in a closed H_2_S-containing atmosphere. For mechanical flexibility tests, silver paste was deposited on two sides of the AgNW TCFs as electrodes. Bending of the AgNW TCFs was performed using a homemade apparatus (extruding AgNW TCFs with two smooth steel blocks), and electrical measurements were performed in real time with the bending.

## 3. Results and Discussion

### 3.1. Influence of FeCl_3_-DS Treatment on the Structure and Optoelectrical Performance of AgNW TCFs

**Figure 1:**
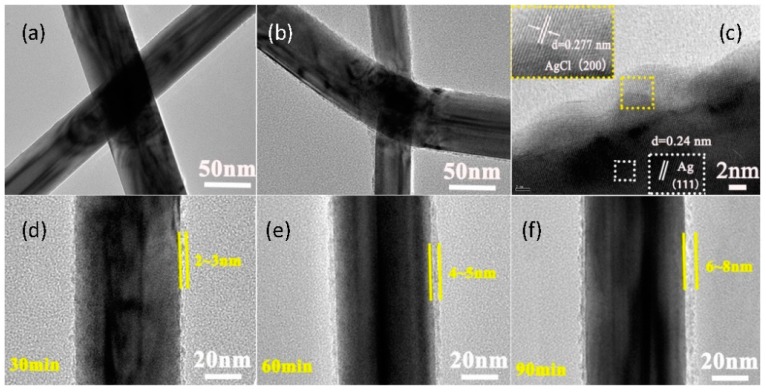
(**a**) TEM image of pristine 50 nm-thick silver nanowires (AgNWs). (**b**) TEM and (**c**) high resolution (HR)TEM images of FeCl_3_ dilute solution (DS) treated 50 nm-thick AgNWs. The inset is the local magnified image of outer AgCl crystals. TEM images of AgNWs treated by FeCl_3_-DS for (**d**) 30 min, (**e**) 60 min, and (**f**) 90 min.

We first examined the structural variation of AgNWs after TCFs were immersed into 0.021 mM FeCl_3_-DS (standard concentration unless specified otherwise) for different time durations. Figure 1a shows the TEM image of pristine 50 nm-thick AgNWs with a smooth surface, and the high resolution TEM (HRTEM) image of a representative AgNW is shown in Figure S1. The nanowires after treatment with FeCl_3_-DS are exhibited in Figure 1b,c. It is clear that the treatment leads to the roughness of the surface of AgNWs. As shown in Figure 1c, lattice fringes of 0.24 nm in the core are indexed to (111) planes of AgNWs, while outer lattice fringes of 0.28 nm can be indexed to (200) planes of cubic-phase AgCl crystals (JCPDS No. 31-1238) [44], which can be further confirmed by the EDS result (Figure S2). These results suggest that a AgCl crystal phase was formed on the surface of AgNWs. By increasing the immersion time, the surface of AgNWs gradually evolved from dispersed particles to a complete layer of AgCl, as shown in Figure 1d–f. The thickness was about 2–3 nm, 4–5 nm, and 6–8 nm for FeCl_3_-DS treatment time of 30 min, 60 min, and 90 min, respectively.

The change of electric properties of the AgNW TCFs during FeCl_3_-DS treatment has been presented in Figure S3. The sheet resistance decreased from 71 Ω/sq to 50 Ω/sq with a decreasing amplitude of 30%. It did not stop decreasing and maintained a relatively stable value until the FeCl_3_-DS treatment time of 90 min. The sheet resistance reduction during aqueous immersion and subsequent drying is caused by the capillary force, which has been thoroughly investigated [37,40], and directly proven here by the same value of sheet resistance for two TCFs in pure water and FeCl_3_-DS, respectively. According to previous studies, the sheet resistance reduction contributed by the capillary force has a limit, so TCFs in pure water for more than 90 min maintain a relatively stable value. However, the sheet resistance increases abruptly by further increasing the immersion time in FeCl_3_-DS to 120 min, under which condition AgNWs with rough surfaces of about 10 nm-thickness are formed, as shown in Figure S4. These results suggest that thick AgCl layers (>10 nm) on AgNWs have a negative effect on the junction conductance between AgNWs. It is worth noting that after a few cycles of 30 min FeCl_3_-DS treatment, the conductivity improvement did not negatively affect the optical performance of AgNW TCFs, as shown in Figure S5.

### 3.2. Endurance of Aging and Both Liquid and Gaseous Chemical Etchings

**Figure 2:**
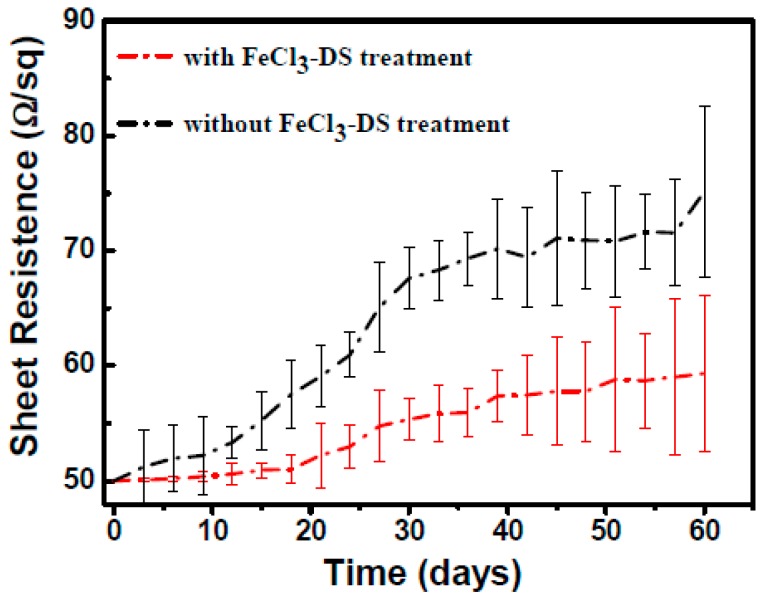
Sheet resistance variation of AgNW transparent conductive films (TCFs) after aging in air for pristine AgNW TCFs (black line) and FeCl_3_-DS treated ones (red line).

We tested the sheet resistance variation of pristine AgNW TCFs and 90 min FeCl_3_-DS treated AgNW TCFs by exposing the films to air. The treated films showed little visual change with aging. As shown in Figure 2, the sheet resistance increased by less than 20% after exposure to air for 60 days. On the contrary, the sheet resistance of the non-treated AgNW TCFs increased by almost 50% after aging for 60 days. Considering that the aging process was in the laboratory environment, the sheet resistance increase may originate from the following processes: (1) AgNWs tend to be oxidized by oxygen or water vapor in the air; (2) AgNWs are prone to sulfurization by trace amounts of H_2_S in air; (3) AgNWs may change when exposed to other chemical vapors. The aging stability of AgNW TCFs improved after a brief FeCl_3_-DS treatment.

**Figure 3:**
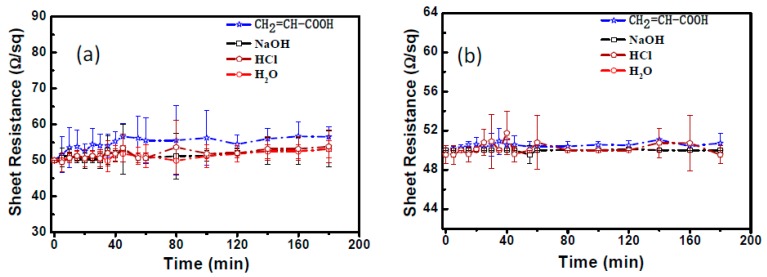
Sheet resistance variation of AgNW TCFs during liquid chemical etching (**a**) without and (**b**) with the 90 min treatment through FeCl_3_-DS.

**Figure 4:**
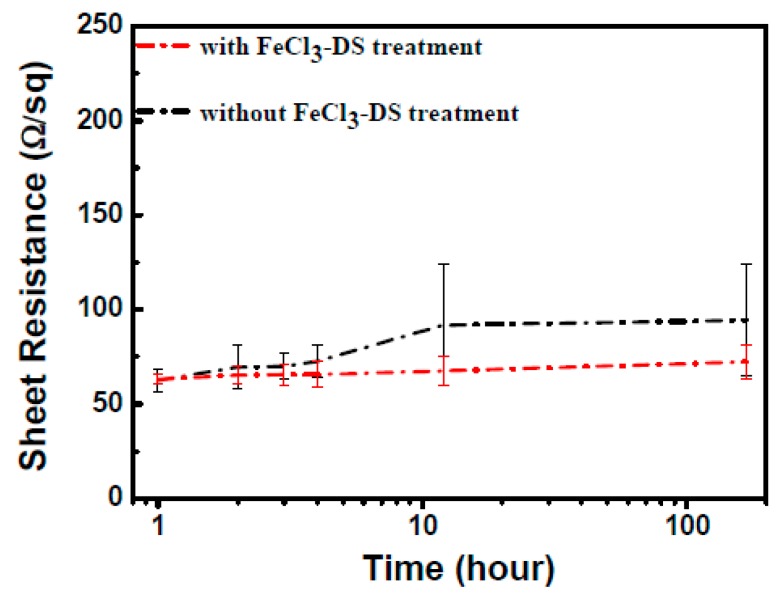
Sheet resistance variation of AgNW TCFs during gaseous chemical etching of H_2_S for pristine AgNW TCFs (black line) and FeCl_3_-DS treated ones (red line).

To carefully study the influence of factors mentioned before, both liquid and gaseous chemical etchings were conducted. The liquid chemical stability was checked by immersing the 90 min FeCl_3_-DS treated AgNW TCFs into solutions of CH_2_=CH-COOH, NaOH, HCl, and deionized water for 180 min, as shown in Figure 3. For non-treated TCFs, the sheet resistance increased quickly up to 15% within 40 min and then slowly stabilized, whereas for FeCl_3_-DS treated ones, the variation was less than 5% after the 3 h duration of solution etchings, which is of great importance in the patterning of AgNW TCFs by solution methods for projected-capacitive touch panel applications. The gaseous chemical stability test was carried out in a closed H_2_S-containing atmosphere. It is observed from Figure 4 that, with increasing sulfurization time up to nearly 200 h, the sheet resistance of the 90 min FeCl_3_-DS treated AgNW TCFs rises slightly. However, the sheet resistance of the non-treated AgNW TCFs increased rapidly up to 50% after the first 12 h of sulfurization. To uncover what happens to AgNWs in a H_2_S-containing atmosphere and the role of FeCl_3_-DS treatment, TEM characterizations were conducted for AgNWs with and without FeCl_3_-DS treatment, as well as before and after gaseous H_2_S etching (Figure S6). From Figure S6b, it is obvious that many tiny nanoparticles form on the surface of AgNWs. Figure S6e shows the HRTEM image of a representative AgNW after the etching of H_2_S, where the lattice fringes of the tiny nanoparticles could be assigned to those of monoclinic-phase Ag_2_S (JCPDS No. 89-3840). Generation of Ag_2_S tiny nanoparticles is further evidenced by both the XPS spectra in Figure S7 and the elemental EDS mapping in Figure S8. However, for 90 min FeCl_3_-DS treated AgNW TCFs, due to the formation of the crystalline AgCl compact layer (Figure 1f, Figures S6c and S9c show the appearance of extra “Cl” peaks in the EDS) as an outer protective layer, the H_2_S attack has no influence on treated AgNW TCFs. No visible changes of the surface morphology could be observed (Figure S6d), and in the EDS of Figure S9d, the amount of Cl shows little change, and no additional “S” peaks could be detected, in striking contrast to the results presented in Figure S9b for unprotected AgNWs in a H_2_S-containing atmosphere. 

Concisely speaking, a brief FeCl_3_-DS treatment largely enhances the stability of AgNW TCFs, whether in aging, liquid, or gaseous chemical etching processes, due to the creation of a thin layer of crystalline AgCl on the surface of AgNWs, observed also by Shin et al. [45]. The detailed mechanism will be carefully studied in the next section.

### 3.3. Endurance of High Temperature: The Mechanism and Inhibition of Size-Dependent Instability

**Figure 5:**
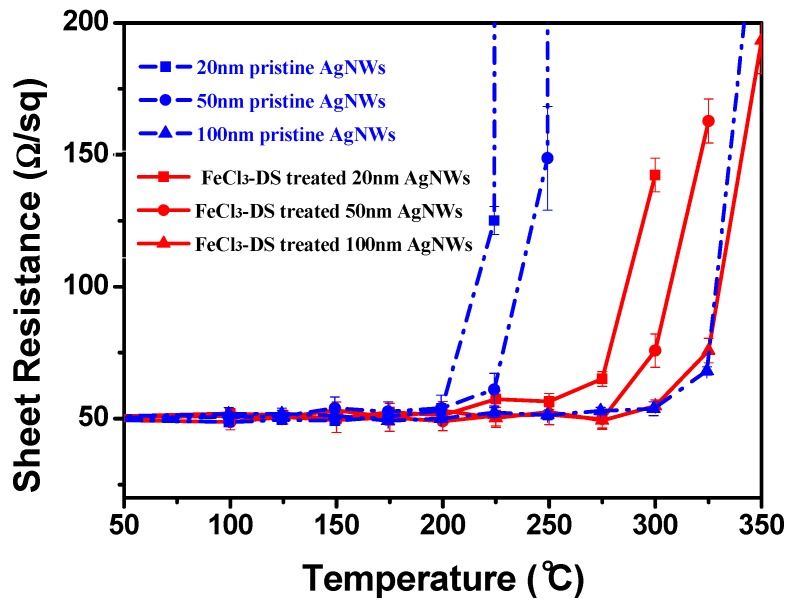
Sheet resistance variation of AgNW TCFs of AgNWs with different diameters in the thermal test: pristine AgNWs (blue lines) and 60 min FeCl_3_-DS treated AgNWs (red lines).

**Figure 6:**
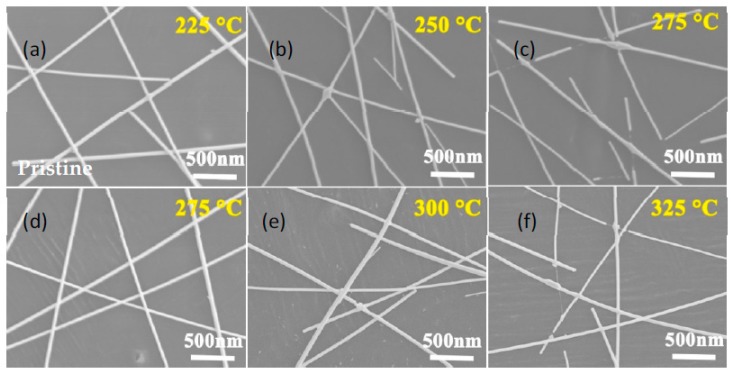
SEM images of pristine and 60 min FeCl_3_-DS treated AgNW TCFs of 50 nm-thick AgNWs under different thermal test temperatures: (**a**) 225 °C, (**b**) 250 °C, and (**c**) 275 °C for pristine AgNWs; (**d**) 275 °C, (**e**) 300 °C, and (**f**) 325 °C for 60 min FeCl_3_-DS treated AgNWs.

The thermal stability was examined by annealing AgNW TCFs at up to 400 °C in a tube furnace in air. Pristine and 60 min FeCl_3_-DS treated AgNW TCFs with different diameters of 20, 50, and 100 nm of AgNWs were considered. As shown in Figure 5, upon annealing, the sheet resistances of pristine AgNW TCFs maintained their initial values until 200 °C, 225 °C, and 300 °C for TCFs of AgNWs with diameters of 20, 50, and 100 nm, respectively. After the turning points, the sheet resistances increased substantially with temperature. It is clear that thicker AgNWs exhibit higher turning point temperatures, which can be explained by the Gibbs–Thomson theory [18]. According to the theory of ΔG = 2γΩ/R, where ΔG is the change in Gibbs–Thomson potential, γ the surface energy, Ω the volume per atom, and R the radius of AgNWs, the AgNW with the larger diameter is subject to a lower potential energy and is therefore comparatively stable compared to the thinner wire. As a result, when temperature rises, the silver atoms in thinner wires first obtain enough thermal energy to diffuse, which, over time, leads to the fragmentation of nanowires. What is exciting is that after a brief FeCl_3_-DS treatment, the turning point temperatures of pristine AgNW TCFs could be increased by 75 °C, from 200 °C to 275 °C, and 225 °C to 300 °C, for AgNWs of 20 nm and 50 nm, respectively. This could not be simply explained by the increase of nanowire diameter, because the diameter increase was less than 10% (4–5 nm/50 nm) for 50 nm-thick AgNWs according to Figure 1e. Actually, the mechanism of thermal instability for AgNWs has been carefully examined by others [46]. The proposed presence of growth waves oriented along the longitudinal axis on the cylindrical surface of AgNWs at elevated temperature creates a periodic stress pattern which drives the surface atomic diffusion from regions of high curvature to those of low curvature [46]. It has been validated here that a conformal surface layer of AgCl through FeCl_3_-DS treatment blocks to some extent the surface Ag atomic diffusion and enhances the turning point temperatures for treated AgNWs, which is vividly presented by the morphology evolution process in Figure 6. For 50 nm-thick AgNWs, as suggested by Figure 5, the pristine TCFs could only endure temperature below 225 °C, so it is found in Figure 6a that AgNWs remain intact in a percolating network. After the temperature rises above the turning point to 250 °C, AgNW junctions begin to fuse together and coalescence into fragmented nanowires (Figure 6b). Further conversion of AgNWs into discrete Ag fragments for pristine AgNWs was observed at 275 °C (Figure 6c). In comparison, as shown in Figure 6d,e, for 60 min FeCl_3_-DS treated AgNW TCFs, AgNWs were found to remain intact in a percolating network until 300 °C. Fragmentation or coalescence of the AgNWs did not happen until the thermal test at 325 °C. The fragmentation was not as serious as that of pristine AgNWs at 275 °C, as shown in Figure 6f. In principle surface Ag atomic diffusion blocking by the conformal layer of AgCl through FeCl _3_-DS treatment endows AgNWs stability during the accelerated aging process in an air environment under a temperature as high as 300 °C. This effect was similar for AgNWs undergoing common aging processes, as well as accelerated aging processes under high temperatures or in a H_2_S-containing atmosphere, and the AgNWs were protected by formation of the physical cap of the AgCl layer to reduce the rate of Ag evaporation. For AgNWs under liquid chemical etching conditions, Ag diffusion in solution instead of evaporation was hindered by the physical cap of the AgCl layer through enhancing the activation energy of surface diffusion [46]. No matter what form it takes effect, a brief FeCl_3_-DS treatment helps AgNW TCFs to withstand aging, both liquid and gaseous chemical etchings, as well as thermal shock for real applications.

**Figure 7:**
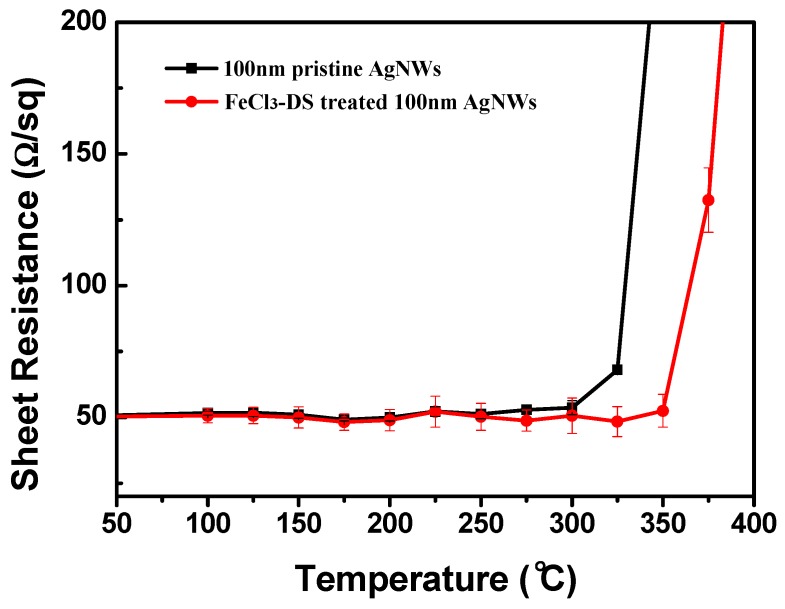
Sheet resistance variation of AgNW TCFs of 100 nm-thick AgNWs in the thermal test: pristine AgNWs (blank line) and AgNWs treated with 0.042 mM FeCl_3_-DS for 120 min (red line).

For 20 and 50 nm-thick FeCl_3_-DS treated AgNWs, the turning point temperatures could be elevated close to that of pristine 100 nm-thick AgNWs, but why did it not change for the 100 nm-thick AgNWs after the standard FeCl_3_-DS treatment (Figure 5)? From careful TEM characterization as shown in Figure S10, it was found that no AgCl layer was formed on the surface of 100 nm-thick AgNWs through the standard FeCl_3_-DS treatment, whereas compact layers existed on the surface of thin AgNWs. The reaction mechanism of AgNWs in FeCl_3_ solution needs in-depth research to disclose the size-dependent formation process of AgCl on the AgNW surface for balancing application requirements between stability and optoelectrical properties through reaction condition adjustment. It is well known that bulk Ag has little chemical reactivity in aqueous ionic solutions, but there have been many reports about the surface oxidation of AgNWs in solutions containing Cl^−^ ions [44,45,47], especially under the help of oxidative Fe^3+^ ions to form AgCl [44]. Then why was no AgCl layer formed on the surface of 100 nm-thick AgNWs through the standard FeCl_3_-DS treatment? Considering the dilute solution of 0.021 mM FeCl_3_, we doubled the concentration and treatment time. As a result, the increase of the turning point temperature eventually occurred by 50 °C for 100 nm-thick AgNWs (Figure 7). Therefore, the reactivity of AgNWs to aqueous ionic solutions is not only related to the size of Ag, but also the adsorbed quantity of ions. As schematically displayed in Figure S11, the redox potential of Ag species is located at −5.239 eV (Ag^+^/Ag vs. vacuum energy), which is 0.028 eV lower than that of Fe species with −5.211 eV (Fe^3+^/Fe^2+^ vs. vacuum energy); therefore, surface oxidation of Ag by Fe^3+^ ions is prohibited. However, for AgNWs, the large surface area helps to adsorb the nucleophilic Cl^−^ ions and this chemisorption could shift the Fermi level of Ag toward vacuum energy by donation of the Cl^−^ ion’s electron density (δe) to surface Ag atoms, whose orbitals are partially occupied [48]. The Fermi level shift could be estimated as follows. The Fermi energy (E_F_), determined by the density of free electrons (N_e_), is expressed as:

$$E_{F}\;=\;(2m/8\pi )(3h{}^{3}N_{e}){}^{2/3}$$

To differentiate on both sides:

$${\Delta E}_{F}\;=\;{(2/3)E}_{F}{(\Delta N}_{e}{/N}_{e})\;=\;{(2/3)E}_{F}f$$

When the density of free electrons is increased by a fraction of f, the Fermi energy is increased by ∆E_F_ through shifting the Fermi level of Ag toward vacuum energy. For AgNWs, the diameter d and length L are much larger than the radius of Ag atoms (r), and each surface atom adsorbs one Cl^−^ ion and contributes one free electron to the electron gas. Then, considering L is 1000 times larger than d,

f = 2r(4/d+2/L)δ » 8rδ/d

$${\Delta E}_{F}\;=\;{(2/3)E}_{F}\left(8r\delta /d\right)\;=\;{(16/3)(E}_{F}r\delta /d)\;=\;(4.68/d)\delta$$

In above equation, E_F_ = 5.48 eV and r = 0.16 nm for Ag. For 100 nm AgNWs, δ with a value above 0.6 could make the Fermi level shift over 0.028 eV, endowing surface Ag atoms in AgNWs with enough reduction ability to be oxidized by Fe^3+^ ions in solution. This means making full use of surface Ag atoms to adsorb a large enough quantity of nucleophilic species. It is also easily understood that for thin AgNWs, a much smaller f and δ could make a large Fermi level shift; while for bulk Ag, though δ reaches its maximum value of 1 and f is at its largest, the Fermi level shift is below 0.01 eV. That is the reason why bulk Ag has little chemical reactivity and why AgNWs could be treated by aqueous ionic solutions, though the treatment is nanowire size-dependent.

**Figure 8:**
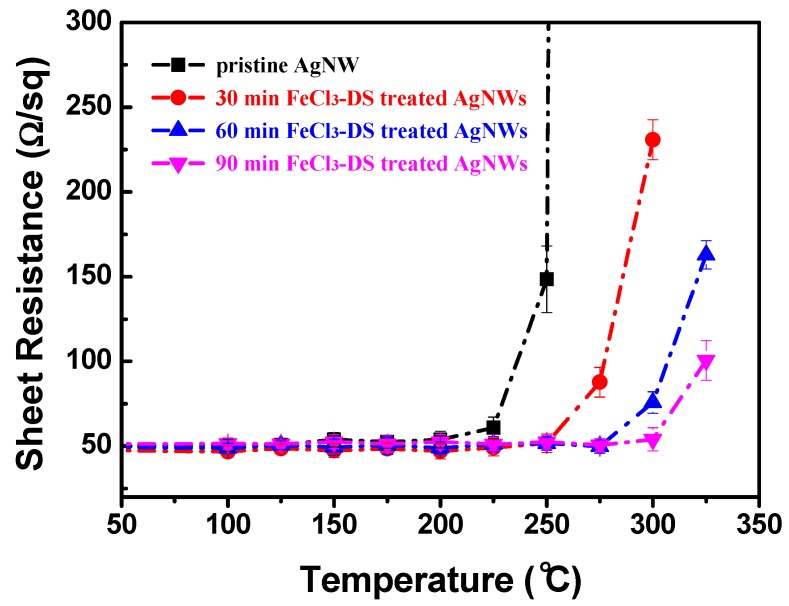
Sheet resistance variation of AgNW TCFs of 50 nm-thick AgNWs in the thermal test: influence of FeCl_3_-DS treatment time.

As the adsorbed quantity of ions has an effect on the reactivity of AgNWs to aqueous ionic solutions, which is used to tune the thickness of the AgCl layer by changing the FeCl_3_-DS treatment time (Figure 1d–f), it should affect the degree of stability enhancement. As shown in Figure 8, the turning point temperature of pristine AgNW TCFs was increased from 225 °C to 275 °C, 300 °C, and 325 °C after FeCl_3_-DS treatment times of 30 min, 60 min, and 90 min, respectively. Is it possible to further increase the turning point temperature by prolonging the FeCl_3_-DS treatment time? Actually, for 50-nm thick AgNWs, 90 min FeCl_3_-DS treatment leads to formation of a compact AgCl layer of 6–8 nm (Figure 1f); further increasing the treatment time deteriorates the conductivity of TCFs as shown in Figures S3 and S12, because when a more than 10 nm-thick AgCl layer is formed, it creates huge junction resistance (Figure S4). Not only treatment time, but FeCl_3_-DS concentration also has major impact on the conductivity of TCFs. As shown in Figure S13, the resistance of TCFs could be decreased by immersing the AgNW films into the dilute FeCl_3_ solution with a concentration below 0.05 mM for a certain time, which has been ascribed to the capillary force of water during drying [37,40]. Yet for FeCl_3_ solutions with concentrations above 0.05 mM, formation of big AgCl particles in quantity will hamper the conductivity of TCFs. It is clearly shown in Figure S14, under a concentration of 1 mM, AgNWs are quickly etched by the FeCl_3_ solution with a huge amount of AgCl particles formed and AgNW films are not conductive at all shortly after immersion. That is the reason why we choose a medium concentration (0.021 mM) of dilute FeCl_3_ solution as the standard one to first enhance the TCF stability by formation of the AgCl layer with a proper thickness; meanwhile, optoelectrical performance (conductance and transparency) should not be impaired. Generally speaking, one has to balance between stability improvement and maintaining optoelectrical performance, especially under the influence of size-dependent instability. Various stabilities of AgNW based hybrid films are compared along with the treatment method, diameter of AgNWs, and transmittance in Table 1.

### 3.4. Mechanical Stability

**Figure 9:**
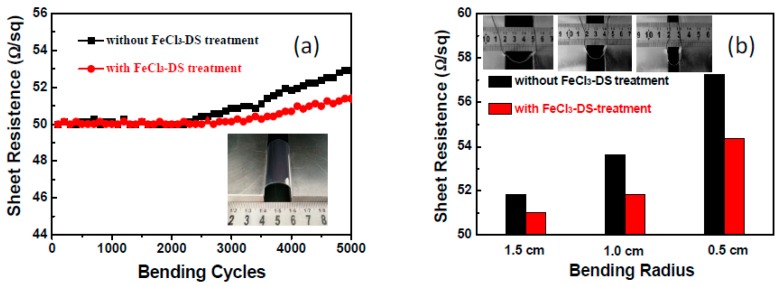
Sheet resistance variation of the films under bending tests (**a**) of bending cycle under a bending radius of 1 cm, and (**b**) with a bending radius of 1.5, 1, and 0.5 cm, respectively, under 5000 bending cycles. The insert in (**a**) is the repeated bending test picture with a bending radius of 1 cm. The insert in (**b**) are the bending test pictures for 5000 times with a bending radius of 1.5, 1, and 0.5 cm.

**Figure 10:**
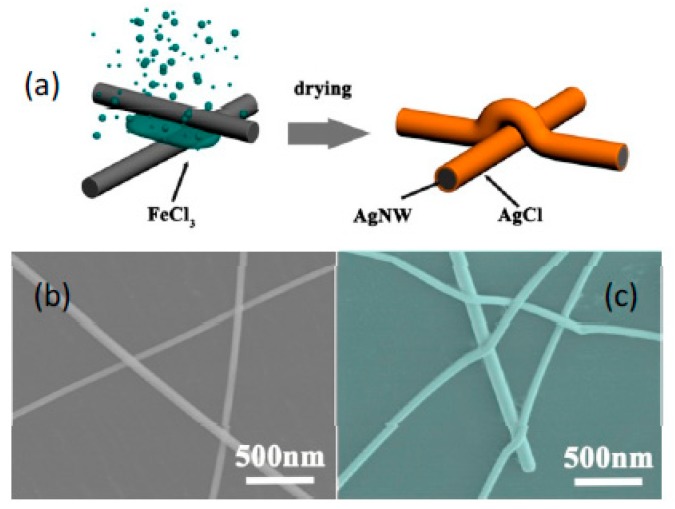
(**a**) Schematic display of FeCl_3_-DS treatment for capillary-force-induced welding of AgNWs and AgCl layer mediated tightening between AgNWs and the substrate as well as among AgNW themselves. SEM images of (**b**) pristine and (**c**) FeCl_3_-DS treated AgNW TCFs.

The most prominent advantage of AgNW TCFs is their flexibility, especially for wearable applications; therefore, the impact of FeCl_3_-DS treatment on the flexibility of AgNW TCFs must be evaluated. Sheet resistances of films with and without FeCl_3_-DS treatment as the function of the bending cycle and bending radius are exhibited in Figure 9. In contrast to degradation, the flexibility was improved by the treatment. As shown in Figure 9a, the sheet resistance of the FeCl_3_-DS treated AgNW TCFs after 5000 bending cycles with a bending radius of 1.0 cm (see inset picture) remained almost unchanged with slight increase of 2.7%. However, the value for the non-treated AgNW TCFs was 5.8% under the same conditions. The sheet resistance increased inversely with the bending radius; even so, the FeCl_3_-DS treated AgNW TCFs exhibited less sheet resistance change than the non-treated ones for all tested bending radii of 1.5 cm, 1.0 cm, and 0.5 cm (Figure 9b). This reveals that the FeCl_3_-DS treatment can increase the mechanical flexibility of the AgNW films due to the welded wire–wire junctions which has been concluded to be induced by capillary force [37,40]. The welded wire–wire junctions can also be induced by the flash light welding process [49,50]. As schematically displayed in Figure 10a, when coating AgNWs on a substrate, pristine AgNWs stack loosely and the physical contacts between nanowires form a nanogap; when AgNW TCFs are exposed to solution and subsequent drying, shrinking occurs preferentially at such nanogaps, leading to the bending of nanowires altogether. The evidence of capillary-force-induced welding of AgNWs is provided by the SEM images shown in Figure 10b,c. The straight and loosely stacked pristine AgNWs bend over each other and wire-wire junctions become tight. Moreover, the conformal formation of the AgCl layer helps to tighten AgNWs to the substrate as well as to the AgNWs themselves, which is clearly evidenced in Figure 10c. Capillary-force-induced welding and AgCl layer mediated tightening are both beneficial for mechanical straining.

Overall, the FeCl_3_-DS treatment not only improves the conductivity and stability of AgNW TCFs, but also enhances their mechanical flexibility.

## 4. Conclusions

We present one simple route to simultaneously improve the stability and flexibility of AgNW TCFs. From comparative experiments of aging, thermal tests, chemical solution etchings, and H_2_S gas etching, the FeCl_3_-DS treated AgNW TCFs show much better stability than pristine films. Moreover, the optoelectrical properties of the AgNW TCFs are not impaired by the FeCl_3_-DS treatment. Making use of size-dependent stability and the chemisorption-related Fermi level shift, different treatments of thin or thick AgNWs for applications in distinct fields are suggested. We believe that this FeCl_3_-DS approach will help to solve the stability issues for practical applications of AgNW TCFs, which have been widely regarded as the next-generation transparent electrodes for various flexible optoelectronics, such as touch panels, displays, smart windows, and so on. Future work will be devoted to further optimization of treatment conditions for long term stability.

## Figures and Tables

**Table 1:** Comparison of AgNW based hybrid films with previous studies.

Protective Materials	Method	AgNW Diameter	T%	Ambient Stability	Thermal Stability	Chemical Stability
GO (Ref. [30])	CVD	~30 nm	↓~5.8%	↑~9% @ 30 days 70 °C/70% RH	/	/
4 nm Ni (Ref. [31])	Electro-deposition	~45 nm	↓~4.5%	↑18% @ 14 days 80 °C/85% RH	/	Yes
5.3 nm Al_2_O_3_ (Ref. [34])	ALD	30 nm	↓~2%	0% @ 45 days 85 °C/85% RH	380 °C/100 min	/
5–15 nm SiO_2_ (Ref. [33])	Dip-coating	36.7 nm	↑~14.3%	/	300 °C/30 min	/
6–8 nm AgCl (This work)	Solution	50 nm	↑~0.4%	↑~16% @ 60 days Lab air	300 °C/20 min	Yes

## Supplementary Materials

The Supplementary Materials are available online at https://www.mdpi.com/2079-4991/9/4/533/s1. Additional SEM, TEM, SAED, XPS and EDS data, the electrical and optical properties of FeCl_3_-DS treated AgNW TCFs and a schematic of Fermi level shift and reaction potentials.
