# Supplementary material for: Tackling the Stability Issues of Silver Nanowire Transparent Conductive Films through FeCl3 Dilute Solution Treatment
Source: Nanomaterials (Basel). 2019 Apr 3;9(4):533. doi: 10.3390/nano9040533 (PMC6523130; doi:10.3390/nano9040533)
Supplement: Supplementary file 1 [file nanomaterials-09-00533-s001.pdf]

Supplementary Materials

Tackling the Stability Issues of Silver Nanowire Transparent Conductive Films through $FeCl_3$ Dilute Solution Treatment

Xikun Chu [1,2,†], Ke Wang [3,†], Jingqi Tao [1,2], Shuxin Li [1], Shulin Ji [1,*] and Changhui Ye [1,4,*]

[1] Key Laboratory of Materials Physics, Anhui Key Laboratory of Nanomaterials and Nanotechnology, Institute of Solid State Physics, Chinese Academy of Sciences, Hefei 230031, China; xikunchu@163.com (X.C.); jqtao127@163.com (J.T.) lishuxin@issp.ac.cn (S.L.)
[2] Science Island Branch of Graduate School, University of Science and Technology of China, Hefei 230026, China
[3] Key Laboratory of Silicon Device Technology, Institute of Microelectronics, Chinese Academy of Sciences, Beijing 100029, China; wangke@ime.ac.cn
[4] College of Materials Science and Engineering, Zhejiang University of Technology, Hangzhou 310014, China
* Correspondence: slji@issp.ac.cn (S.J.); chye@zjut.edu.cn (C.Y.); Tel.: +86-551-65591923 (S.J.)
† These authors contributed equally to this work.

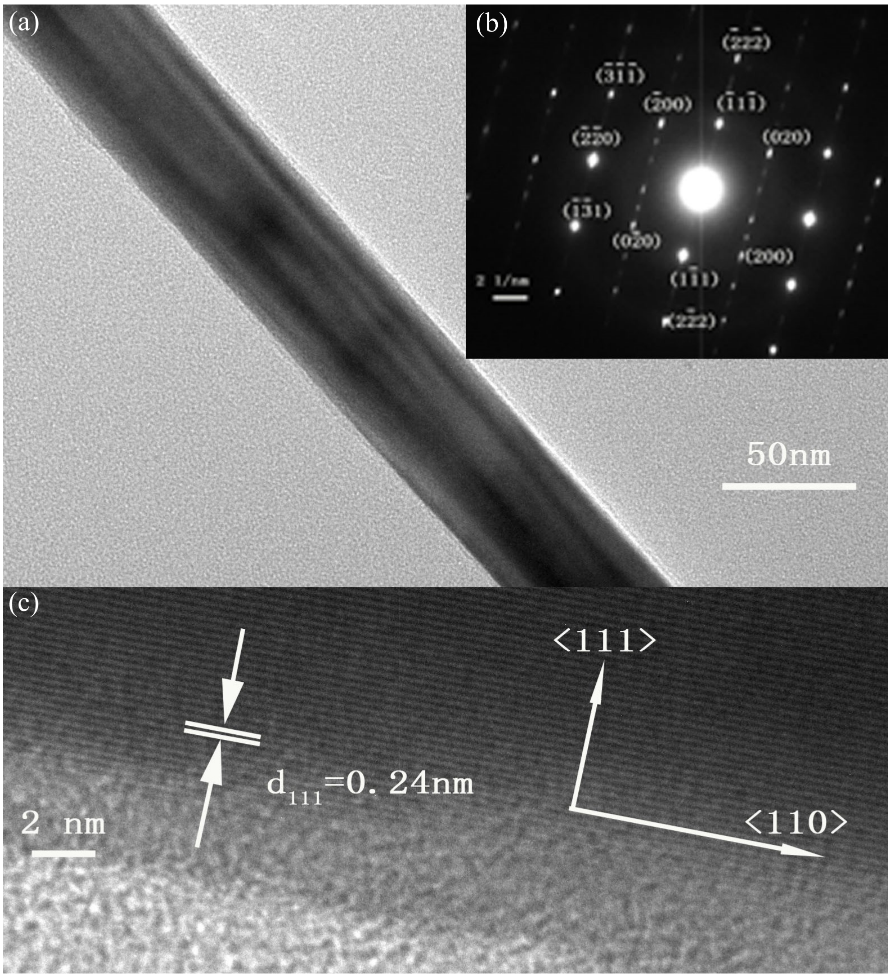

Figure S1. (**a**) TEM image, (**b**) corresponding selected area electron diffraction (SAED) pattern and (c) high resolution TEM image of a representative single AgNW.

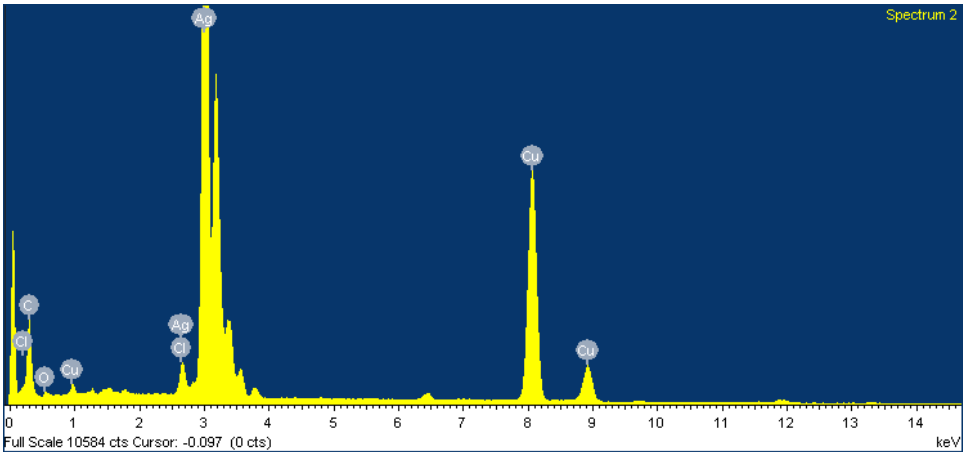

Figure S2. An EDS spectrum of AgNWs after the treatment through $FeCl_3$-DS.

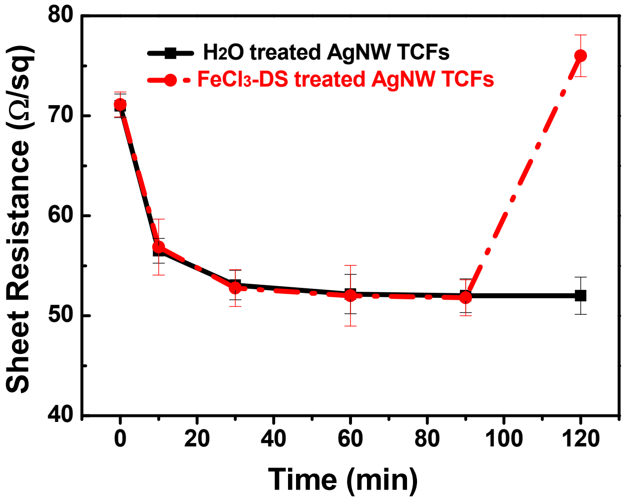

Figure S3. Sheet resistance change of AgNW TCFs of 50 nm-thick AgNWs as a function of the immersion time in $FeCl_3$-DS and H_2O.

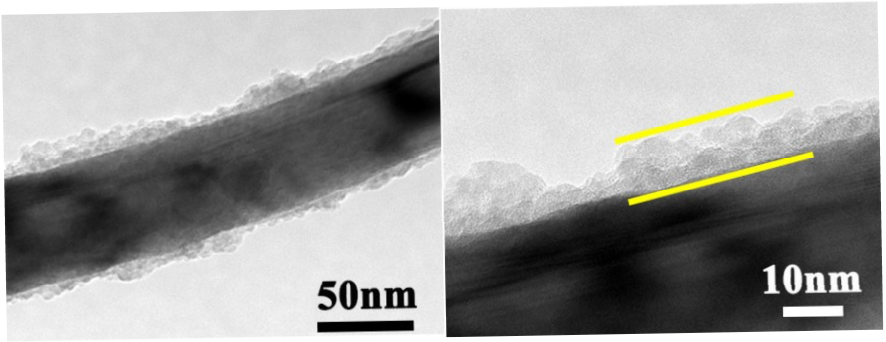

Figure S4. TEM images of 50 nm-thick AgNWs treated by $FeCl_3$-DS for 120 min.

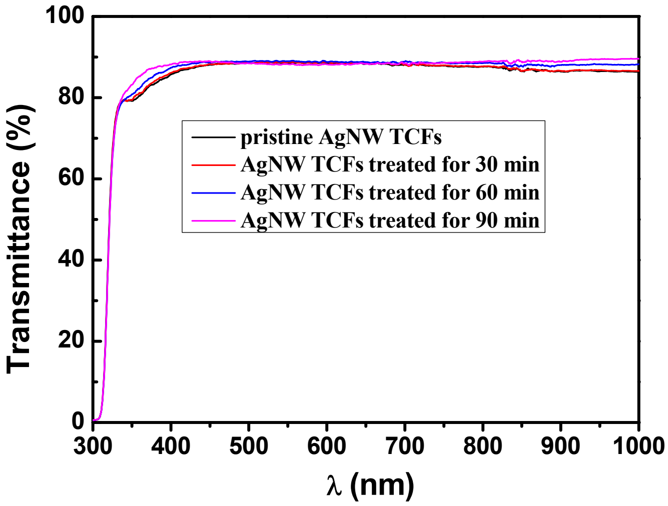

Figure S5. Total transmittance of pristine AgNW TCFs (black line) as well as 30 min, 60 min and 90 min $FeCl_3$-DS treated AgNW TCFs.

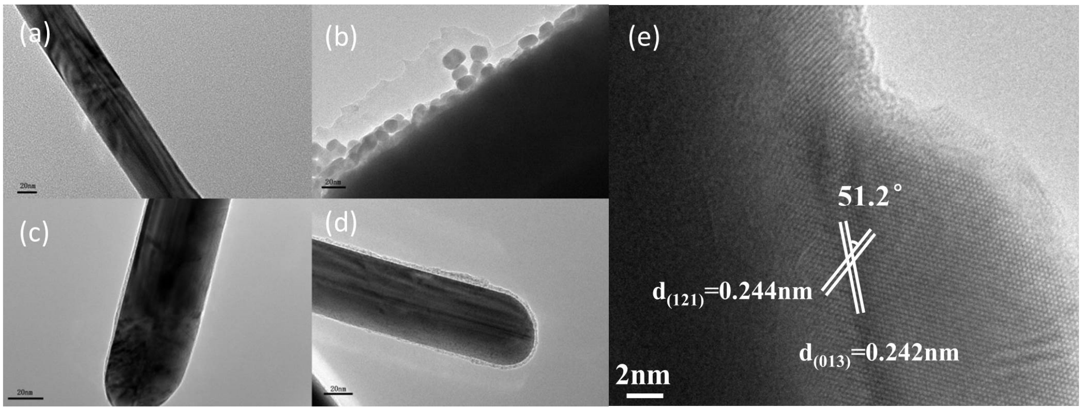

Figure S6. TEM images of pristine AgNWs (**a**) before and (**b**) after H_2S etching. TEM images of 90 min $FeCl_3$-DS treated AgNWs (**c**) before and (**d**) after H_2S etching. (**e**) HRTEM image of a representative unprotected AgNW after the etching of H_2S.

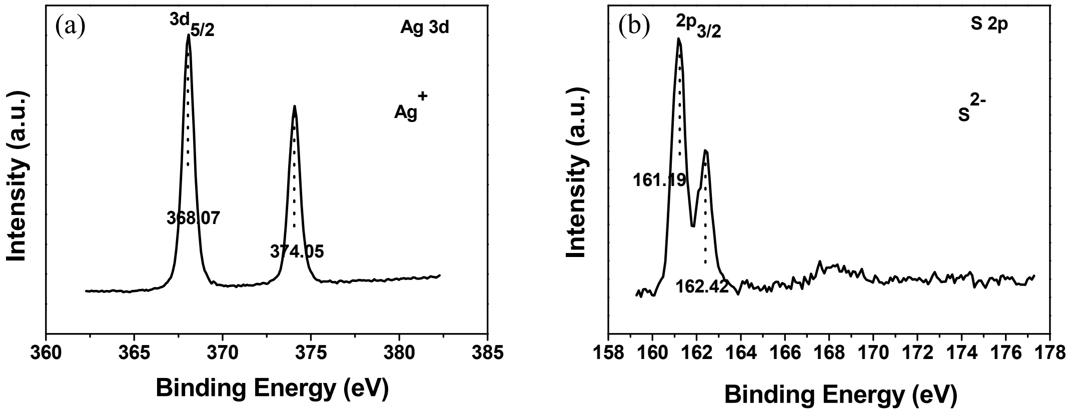

Figure S7. The X-ray photoelectron spectra of unprotected AgNW TCFs after the etching of H_2S: (a) Ag 3d, (b) S 2p.

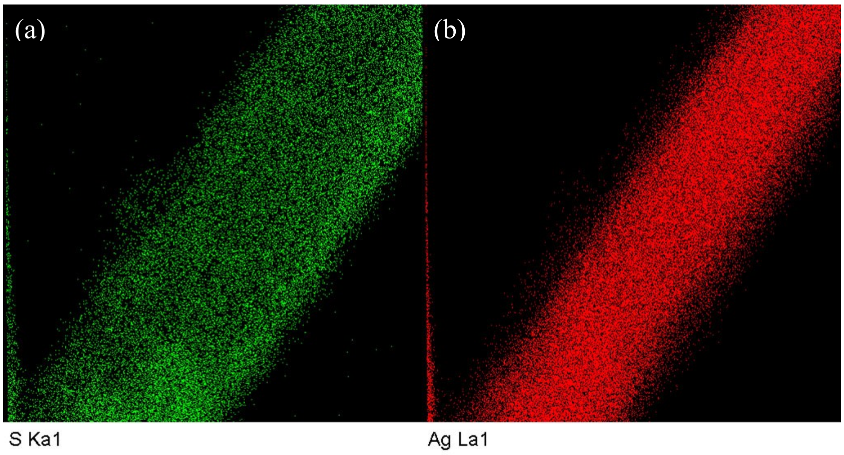

Figure S8. EDS mapping of unprotected AgNW TCFs after the etching of H_2S.

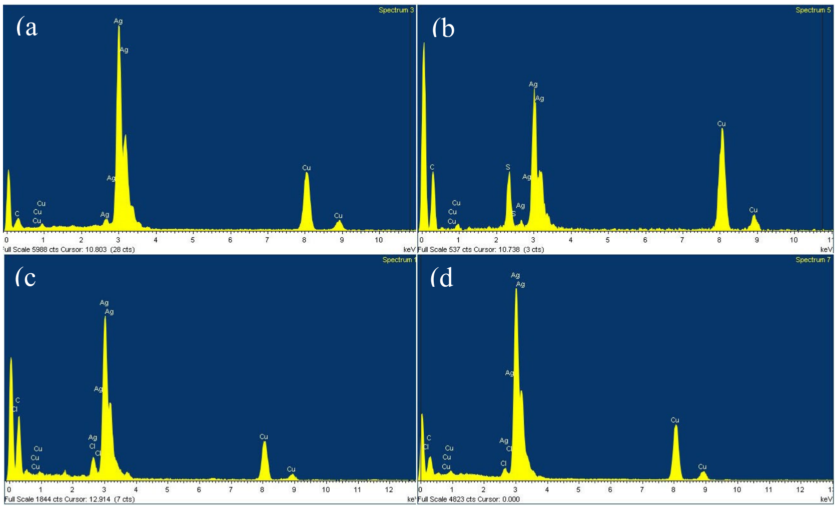

Figure S9. EDS spectra of pristine AgNWs (**a**) before and (**b**) after the etching of H_2S. EDS spectra of 90 min $FeCl_3$-DS treated AgNWs (**c**) before and (**d**) after the etching of H_2S. (Element Cu is derived from the substrate copper network for TEM)

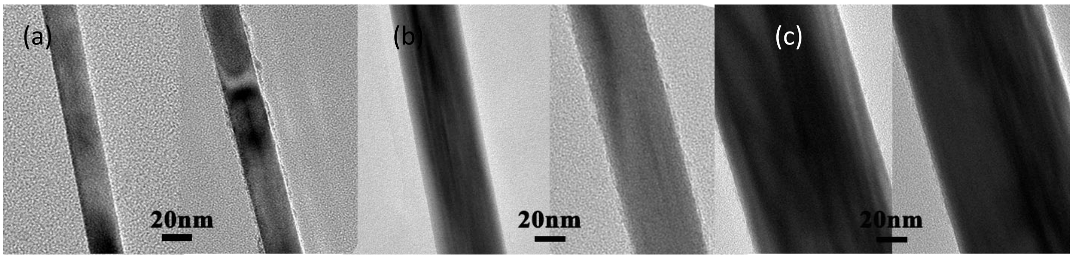

Figure S10. TEM images of pristine and corresponding 60 min $FeCl_3$-DS treated AgNWs of different diameters: (**a**) 20 nm, (**b**) 50 nm and (**c**) 100 nm.

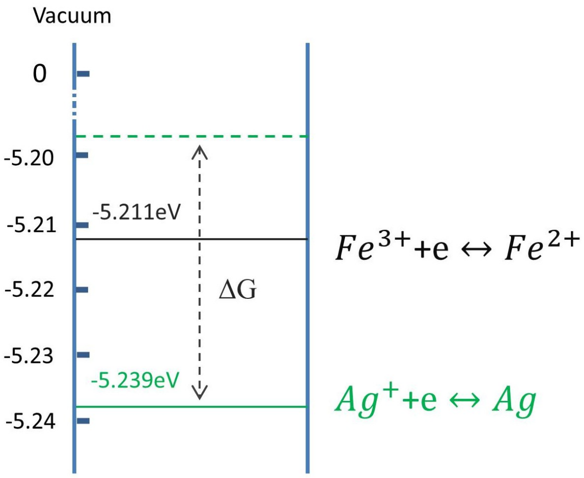

Figure S11. Schematic display of energy levels of reactions in solution and Fermi level shift of Ag due to chemisorption of Cl^- ions.

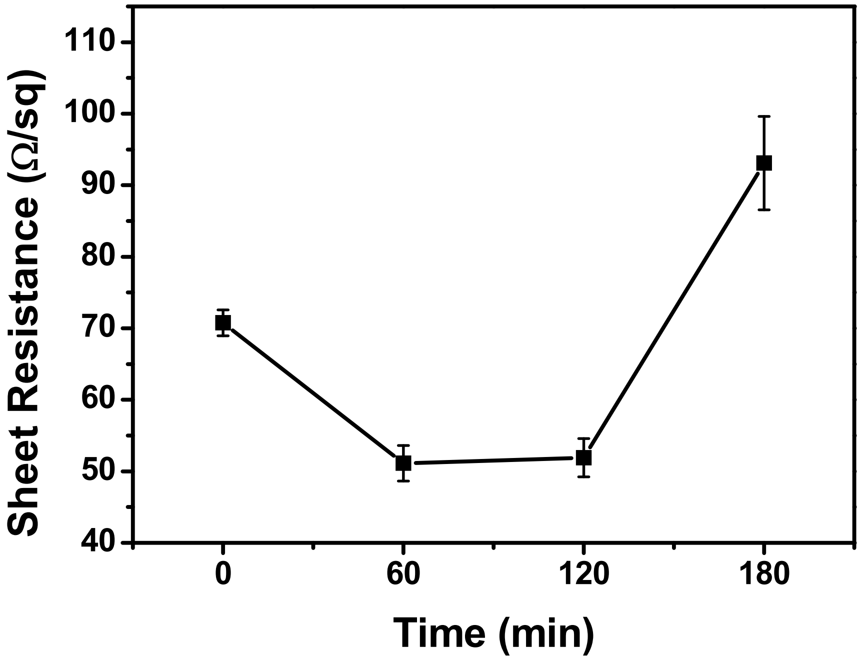

Figure S12. Sheet resistance change of AgNW TCFs of 100 nm-thick AgNWs as a function of the immersion time in 0.042 mM $FeCl_3$-DS.

(a)

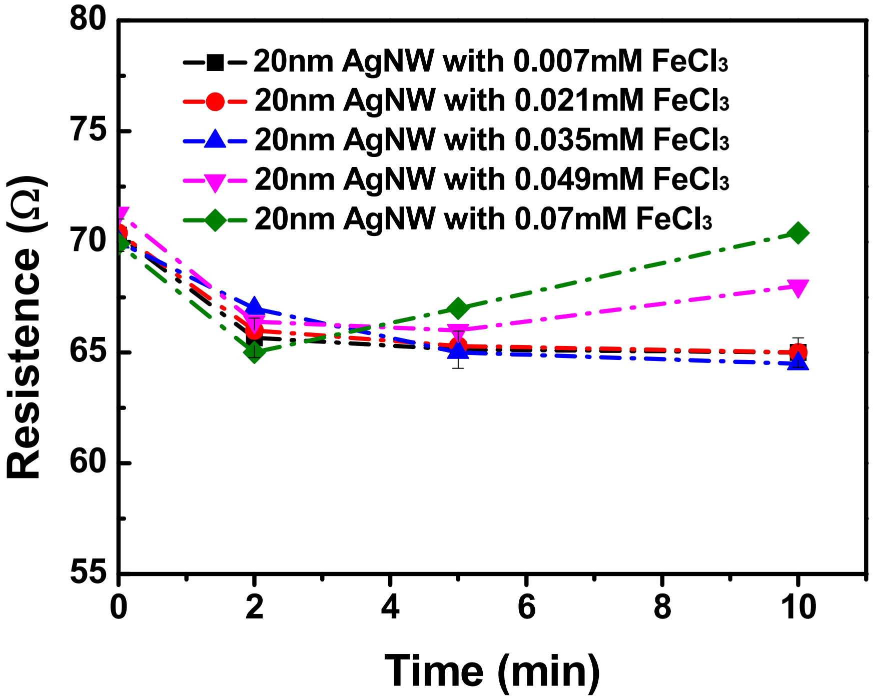

(b)

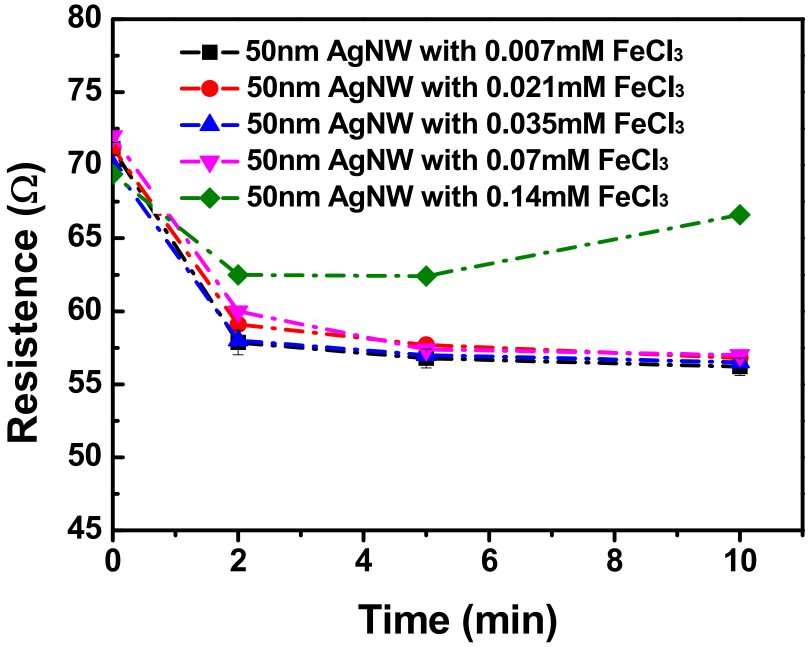

Figure S13. Resistance change of AgNW TCFs as a function of the immersion time in $FeCl_3$-DS under different concentrations for: (**a**) 20 nm-thick AgNWs and (**b**) 50 nm-thick AgNWs.

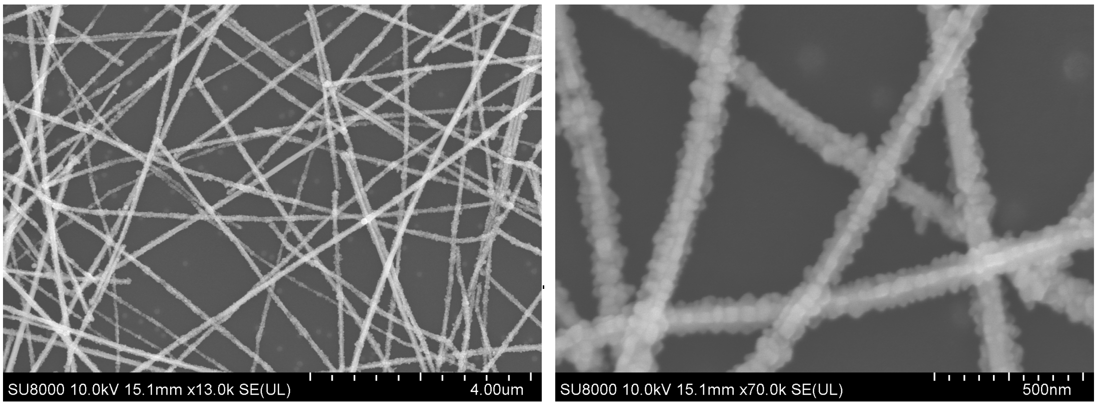

Figure S14. SEM images of 50 nm-thick AgNWs treated shortly by 1 mM $FeCl_3$ solution.
